# The effect of embryo transfer technique on pregnancy rates in *in vitro* fertilization-intracytoplasmic sperm injection cycles: A prospective cohort study

**DOI:** 10.4274/tjod.galenos.2021.03073

**Published:** 2021-03-12

**Authors:** Zeynep Öztürk İnal, Hasan Ali İnal

**Affiliations:** 1 Konya Training and Research Hospital, Clinic of Reproductive Endocrinology, Konya, Turkey

**Keywords:** Embryo transfer, infertility, clinical pregnancy rate, assisted reproductive technology

## Abstract

**Objective:**

To investigate whether embryo transfer affects pregnancy rates in *in vitro* fertilization-intracytoplasmic sperm injection (IVF-ICSI) treatment.

**Materials and Methods:**

A total of 2,257 patients who underwent IVF-ICSI treatment between 2012 and 2017 were included in this study. Subjects were categorized according to the embryo transfer technique that was required: group 1 (n=1,657) underwent easy transfer with a soft catheter; group 2 (n=548) received external guidance transfers; and group 3 (n=52) experienced difficult transfers with a stylet. Basal parameters, clinical and laboratory IVF-ICSI outcomes, and clinical pregnancy rates (CPR) were compared between the groups.

**Results:**

There were no differences between the groups in terms of age, body mass index, smoking status, duration and etiology of infertility, baseline folliclestimulating hormone, luteinizing hormone, estradiol (E_2_), thyroid-stimulating hormone, prolactin levels, antral follicle count, duration of stimulation, stimulation protocol, total gonadotropin dose required, peak E_2_ levels, progesterone levels, and endometrial thickness on human chorionic gonadotropin administration and transfer days (p>0.05). The numbers of oocytes retrieved, MII and 2PN, fertilization rate, day of embryo transfer, and CPRs were also comparable between the groups (p>0.05).

**Conclusion:**

Our data suggest that embryo transfer has no impact on pregnancy rates in patients who undergo IVF-ICSI treatment. Further studies with more participants are required to elucidate this situation.

**PRECIS:** Embryo transfer technique has no impact on pregnancy rates in patients who have undergone IVF-ICSI treatment.

## Introduction

Despite all the developments in assisted reproductive technology (ART) since the first live birth following *in vitro *fertilization (IVF) in 1978, pregnancy rates have remained at around 35-45%^(1,2,3,4)^. In ART cycles, the method of embryo transfer (ET) is important to clinical pregnancy success in addition to features such as age, the endometrial receptivity of the infertile woman, and embryo quality^(5,6,7,8)^. It has been claimed that faulty ET is responsible for 25-30% of failed implantations, relating either to the catheter application technique or the experience of the physician performing the ET procedure^(9,10,11,12)^.

It has also been asserted that performing mock ET before IVF-intracytoplasmic sperm injection (ICSI) can improve the success of the real ET^(9)^, but studies have also shown that a mock procedure does not reflect all problems that may occur during the actual ET^(5,9)^. Consequently, afterloading ET with external guidance has been suggested to avoid problems that may be encountered^(5,11)^. In the afterloading technique, a catheter with an inner sheath is inserted into the external cervical os, passed through the cervical canal to exit the internal os, and then advanced to 10 mm of the uterine fundus by gentle movement and guided by ultrasonography (USG). A second catheter with embryo-loaded inner sheath is inserted along the same pathway from the first retracted catheter and advanced to 10 mm of the uterine fundus, and the embryos are released into the endometrium^(11)^. The process is intended to minimize embryonic and endometrial trauma^(5)^.

In this study, we aimed to investigate whether the ET technique used affects the pregnancy rates of patients who undergo IVF-ICSI treatment.

## Materials and Methods

### Study Participants and Data Collection

A prospective cohort study was conducted at Ali Kemal Belviranlı Women’s Health and Children’s Hospital, IVF Unit. Outcomes of 2257 fresh ICSI cycles were included consecutively between January 2012 and December 2017. Women were included in the study if they were aged 20-44 years. All of the patients had a body mass index (BMI) between 18 and 35 kg/m^2^, regular menstrual cycles, no uterine abnormalities in an ultrasound examination, and normal baseline hormonal levels. Participants were excluded from the study if they were aged ^>^45 years, BMI ^>^35 kg/m^2^, any significant illness or metabolic disorders. Ethics board approval was given from the institutional review board (2012/57). Written and oral informed aggrement was given from the participants.

Data were obtained for age, BMI (kg/m^2^), smoking status, infertility period, cause of infertility, the baseline at day 3 for follicle-stimulating hormone (FSH), luteinizing hormone (LH), and estradiol (E_2_) levels, thyroid-stimulating hormone (TSH), prolactin, antral follicle count, stimulation parameters, IVF-ICSI outcomes, and clinical pregnancy rates (CPR).

### Ovarian Stimulation and Oocyte Retrieval

Controlled ovulation stimulation was negotiated using the gonadotropin-releasing hormone agonist (GnRHa) or the flexible gonadotropin-releasing hormone antagonist (GnRHant) protocol.

**The GnRHa Protocol:** First, pituitary down-regulation was achieved with a GnRH agonist. Then, the exogenous gonadotropins (Puregon; Organon, Oss, the Netherlands, or Gonal F; Serono, Istanbul, Turkey) were used for ovarian stimulation. The GnRH agonist leuprolide acetate (Lucrin; Abbott Cedex, Istanbul, Turkey) was preferred subcutaneously daily from day 21 of the preceding luteal phase (0.5 mg/day, sc) until menstruation, and then the dose was decreased to 0.25 mg/day until ovulation was triggered. The initial gonadotropin dose used for ovarian stimulation was started according to the patient’s age, baseline serum FSH concentrations on day 3, BMI, and previous response to ovarian stimulation. The starting regimen was fixed for the first 3 days (100-225 IU recombinant FSH/day). Thereafter, the dose of gonadotropin was adjusted according to the individual ovarian responses, which were monitored by measuring serum E_2_ levels and transvaginal USG (LOGIC 200 PRO, GENERAL ELECTRIC, Seoul, South Korea). The administration of 250 IU recombinant human chorionic gonadotropin (hCG) (Ovitrelle, Serono, Istanbul, Turkey) was preferred for the ovulation triggering when at least two follicles reached 18 mm in diameter. Oocytes were retrieved 36 h after the hCG injection, and ICSI was performed for all patients undergoing IVF-ET.

**Microdose Flare-up Protocol:** Recombinant FSH (Puregon; Organon, Oss, the Netherlands, or Gonal F; Serono, Istanbul, Turkey) and the GnRH agonist leuprolide acetate daily together (Lucrin; Abbott Cedex, Istanbul, Turkey) were administered subcutaneously (0.5 mg/day, subcutaneously for 5 days) on day 3 of a withdrawal bleed following at least 3 weeks of oral contraceptive use. The initial gonadotropin dose used was individualized according to the patient’s age, baseline serum FSH concentration on day 3, BMI, and previous response to ovarian stimulation. The starting regimen was fixed for the first 3 days (100-225 IU recombinant FSH/day). Thereafter, the dose of gonadotropin was adjusted according to the individual ovarian responses, which were monitored by measuring serum E_2_ levels and transvaginal USG (LOGIC 200 PRO, GENERAL ELECTRIC, Seoul, South Korea). Ovulation was triggered by the administration of 250 IU recombinant hCG (Ovitrelle, Serono, Istanbul, Turkey) when at least two follicles reached 18 mm in diameter. Oocytes were retrieved 36 h after the hCG injection, and ICSI was performed for all patients undergoing IVF-ET.

**The GnRHant Protocol:** The flexible GnRHant protocol was used for the pituitary down-regulation. Recombinant human FSH (r-FSH; Gonal-F, Merck-Serono, or Puregon, MSD) or human menopausal gonadotropin (hMG; Menogon or Menopur; Ferring) was preferred for COH. The initial gonadotropin dose used for ovarian stimulation was started according to the patient’s age, baseline serum FSH concentrations on day 3, BMI, and previous response to ovarian stimulation. The starting regimen was fixed for the first three days (150-225 IU rec FSH/day), and thereafter, the gonadotropin dose was adjusted according to the individual’s ovarian response. Serial estrogen levels were measured and two-dimensional follicle measurements by transvaginal USG (LOGIC 200 PRO, GENERAL ELECTRIC, Seoul, South Korea) were assessed. A daily dose of 0.25 mg of GnRHant (Cetrotide, Merck-Serono, or Orgalutran, MSD) was started when the leading follicle diameter was ≥13 mm or the serum E_2_ level reached ≥300 pg/mL. When at least two dominant follicles reached dimensions of 18 mm or greater in diameter, hCG (250 µg, Ovitrell, Merck-Serono) was administered, and oocytes were retrieved 36 hours after the hCG injection. ICSI was then applied following our clinical procedures.

### ET Procedure

All ETs were performed with a full bladder under USG guidance (Logiq 200 Pro, General Electric, Seoul, South Korea) using an ET catheter system (Rocket Genesis R57630-00-23 and R57591-00-23). The degree of difficulty of each ET was determined according to the opinion of two physicians as either easy (with a soft catheter), moderate (with external guidance), or difficult (with stylet). With the patient in the lithotomy position, a sterile speculum was introduced to the vagina and the cervix visualized. If mucus or other debris were present, these were cleared using sterile cotton swabs.

An embryologist loaded the embryos into a soft transfer catheter, which was passed to the ET physician who deposited the embryos approximately 10 mm from the uterine fundus under USG guidance. The catheter was gently removed after 5 seconds. In cases of ET with external guidance, an initial catheter with an inner sheath was inserted into the external cervical os, and then advanced through the cervical canal and internal os to 10 mm of the uterine fundus using USG. The internal sheath was withdrawn, and a second catheter loaded with embryos was introduced in its place and advanced to approximately 10 mm from the uterine fundus where the embryos were deposited. “Difficult transfer” was defined if the use of a stylet was required in addition to this form of external guidance.

All catheters were immediately flushed with media to check for embryos, blood, or mucus, and the patient remained in the Trendelenburg position for about 10 minutes. Patients in whom tenaculum was used were excluded from the study. Progesterone in the form of Crinone 8% gel (Serono, Istanbul, Turkey) at a daily dosage of 90 mg for 14 days was given for luteal phase support. Subjects were categorized according to the ET technique required: group 1 (n=1657) experienced an easy transfer with the soft catheter; group 2 (n=548) required afterloading external guidance; and group 3 (n=52) underwent a difficult transfer using a stylet. Basal parameters, clinical and laboratory IVF-ICSI outcomes, and pregnancy rates were compared between the groups.

### Statistical Analysis

Statistical analyses were performed using the SPSS 15.0 for Windows software package (SPSS, Chicago, IL, USA). The Shapiro-Wilk test was used for examining the continuous variables with normal and abnormal distributions, and One-Way analysis of variance (ANOVA) was used for normally distributed continuous variables. The Kruskal-Wallis test was used for abnormally distributed continuous variables. When the Kruskal-Wallis test indicated statistically significant differences, the causes of those differences were determined using a Bonferroni-adjusted Mann-Whitney U test. Categorical data were analyzed using Pearson’s chi-square test, and Fisher’s Exact test was used if the expected frequency was less than 5 in >20% of all cells. Continuous variables are presented as mean ± standard deviation and categorical variables are presented as the number of cases and percentages. For all possible multiple comparisons, Bonferroni-adjustment was performed to control for type I errors. Statistical significance was accepted at p<0.05.

The sample size calculation was performed using the DSS statistical software package for research sample size calculations ^(13)^. The primary aim of this study was to compare the differences in CPR between the groups. It was calculated that a minimum of 50 participants in each group would be required to demonstrate a difference of at least 10% between the groups, with a power of 80% at the 5% significance level. This difference of 10% was taken both from a pilot study and our clinical experiments.

## Results

**Figure 1:**
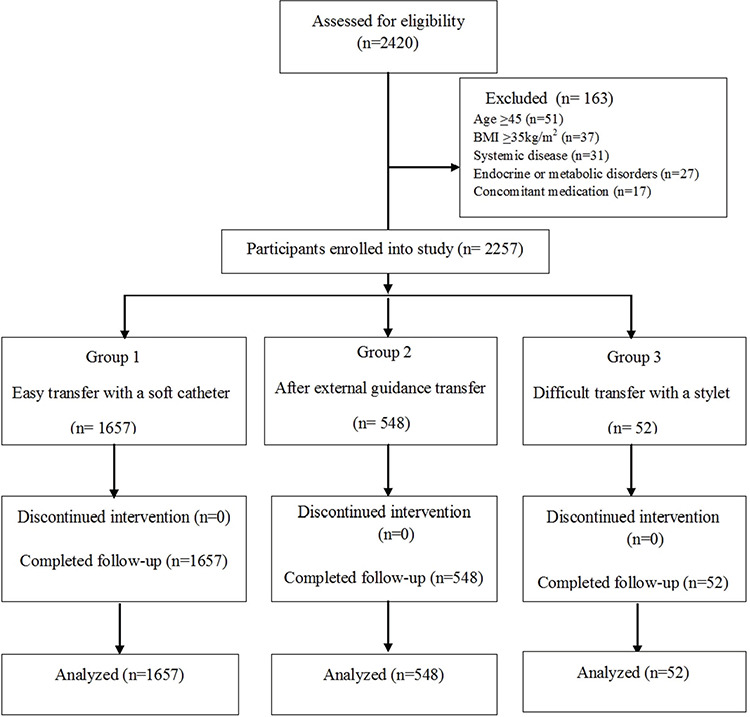
Enrollment and follow-up of the study subjects

A total of 163 patients were excluded from the study, specifically those with aged ^>^45 (n=51), BMI ^>^35 kg/m^2^ (n=37), systemic disease (n=31), endocrine or metabolic disorders (n=27), and concomitant medication (n=17). The remaining 2,257 patients were classified into the three ET groups and their outcomes analyzed (Figure 1).

A comparison of the sociodemographic and stimulation characteristics of the participants is provided in Table 1. There were no differences between the groups in terms of age, BMI, smoking status, duration and etiology of infertility, baseline FSH, LH, E_2_, TSH, prolactin levels, antral follicle count, duration of stimulation, stimulation protocol, total gonadotropin dose required, peak E_2_ levels, progesterone levels, and endometrial thickness on hCG administration and transfer days (p>0.05).

The laboratory and reproductive outcomes of the participants are summarized in Table 2. The numbers of oocytes retrieved, MII and 2PN, fertilization rate, the day of ET, and the CPR were comparable between the groups (p>0.05).

## Discussion

The current study aimed to investigate whether the ET technique required during IVF-ICSI treatment affected pregnancy rates. We found no such impact in our study population.

Some existing studies have shown that the type of ET used can have as significant an effect on pregnancy rates in IVF-ICSI cycles as clinical and embryologic features, but different results have been reported^(5,6,7,8,9,10)^. In particular, Yılmaz et al.^(5)^ retrospectively compared the effects of undergoing easy, moderate, or difficult ET on CPR in 313 IVF-ICSI cycles and found that difficult ET was associated with lower CPR but with no statistically significant difference. Elsewhere, Burke et al.^(14)^ compared 159 cases of fresh ET with 46 frozen ETs and found that the technique and type of catheter used did not affect CPR. Cervical dilatation was performed under general anesthesia in IVF-ICSI cycles of 57 patients who had previously experienced difficult ET; easy ET was performed with a soft catheter in 70% of the participants with the remaining 30% experiencing difficult transfer, and an increased pregnancy rate was observed in those who underwent soft catheter ET^(15)^. Similarly, a meta-analysis of 23 randomized controlled trials reported that ET performed with soft catheters had an overall positive effect on CPR, with the authors suggesting this was due to minimizing uterine contractions^(16)^.

In a retrospective analysis comparing easy and difficult ET with a total of 7,714 ETs included, CPR was found to be significantly higher in the easy ET group^(17)^. In this study, the authors classified direct ET that did not require any intervention as easy ET, and ET that required afterloading external guidance as difficult. They claimed the possible reasons for low CPR in difficult ET as endometrial lesions and the induction of uterine contractions caused by the intervention. However, when they performed subgroup analysis, no statistically significant difference was found in terms of CPR in the group requiring easy ET and afterloading external guidance, only ET duration was found longer in the intervention group.

In contrast, Sallam et al.^(18)^ evaluated 784 IVF cycles, including 655 IVF-ICSI treatments, and found that negotiation of the cervix, the use of a vulsellum, and the presence of blood on the catheter wall or cervix did not affect CPR. Neithard et al.^(11)^ reported that ET performed with afterloading external guidance increased pregnancy rates when compared with difficult ETs, but this increase did not reach a statistically significant level in their retrospective evaluation of 127 IVF-ICSI cycles. It was also observed that blood and mucus were present in the catheters of the difficult ET group, which the authors suggested could stimulate uterine contractions and negatively affect embryos. These negative conditions, the paper claimed, could have been prevented by using ET with external guidance.

Possible confounding factors on CPR, such as the presence of blood or mucus, were excluded in the current study. Nevertheless, there was no difference between the groups in terms of possible confounding factors that could have affected CPR, and we consequently explored ET type alone and found that it did not affect CPR.

### Study Limitations

The strengths of the present study include its prospective design, the sufficient number of participants in each group, and the representative sample from central Turkey. The results of the study can be generalized to the majority of the country’s population. Another strength is that the same two senior physicians performed all ETs, which helps minimize external human effects on CPR. However, the potential weaknesses of the study are that it was conducted in a tertiary care institution and that the cumulative CPR was not evaluated because no frozen ETs were included.

## Conclusion

Our data showed that there was no impact of ET technique on the pregnancy rates of these patients who underwent IVF-ICSI treatment. Further studies with more participants are required to elucidate this situation.

## Figures and Tables

**Table 1:**
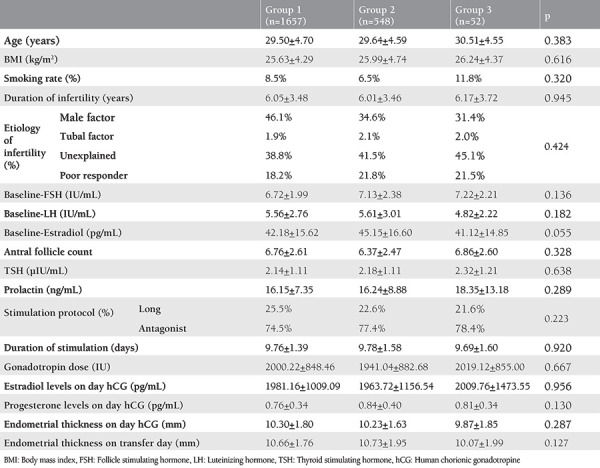
Demographic and stimulation characteristics of the patients

**Table 2:**
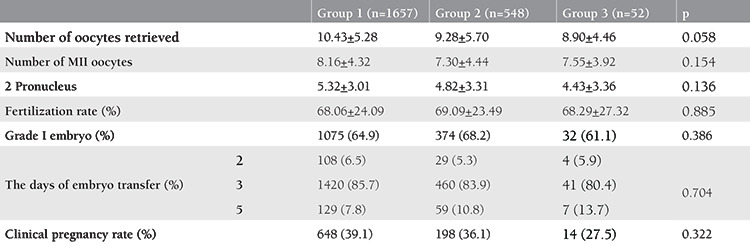
Laboratory and reproductive outcome parameters of the patients
